# Asian-Origin Approved COVID-19 Vaccines and Current Status of COVID-19 Vaccination Program in Asia: A Critical Analysis

**DOI:** 10.3390/vaccines9060600

**Published:** 2021-06-04

**Authors:** Chiranjib Chakraborty, Ashish Ranjan Sharma, Manojit Bhattacharya, Govindasamy Agoramoorthy, Sang-Soo Lee

**Affiliations:** 1 Department of Biotechnology, School of Life Science and Biotechnology, Adamas University, Barasat-Barrackpore Rd, Kolkata 700126, West Bengal, India; 2 Institute for Skeletal Aging & Orthopedic Surgery, Hallym University-Chuncheon Sacred Heart Hospital, Chuncheon-si 24252, Gangwon-do, Korea; boneresearch@hallym.ac.kr; 3 Department of Zoology, Fakir Mohan University, Vyasa Vihar, Balasore 756020, Odisha, India; mbhattacharya09@gmail.com; 4 College of Pharmacy and Health Care, Tajen University, Yanpu, Pingtung 907, Taiwan; agoram@tajen.edu.tw

**Keywords:** Asia, COVID-19 vaccines, COVID-19 vaccination, factors infusing COVID-19 vaccination

## Abstract

COVID-19 vaccination has started throughout the globe. The vaccination program has also begun in most Asian countries. This paper analyzed the Asian-origin COVID-19 vaccines and vaccination program status in Asia till March 2021 under three sections. In the first section, we mapped the approved vaccines that originated from Asia, their technological platforms, collaborations during vaccine development, and regulatory approval from other countries. We found that a total of eight Asian COVID-19 vaccines originated and got approval from three countries: China, India, and Russia. In the second section, we critically evaluated the recent progress of COVID-19 vaccination programs. We analyzed the overall vaccination status across the Asian region. We also calculated the cumulative COVID-19 vaccine doses administered in different Asian countries, vaccine rolling in 7-day average in various Asian countries, and COVID-19 vaccine per day doses administrated in several Asian countries. We found that China and India vaccinated the maximum number of people. Finally, we evaluated the factors affecting the COVID-19 vaccination program in Asia, such as vaccine hesitancy, basic reproduction numbers (R0) and vaccination campaigns, and the cost of the vaccines. Our analysis will assist the implementation of the COVID-19 vaccination program successfully in Asia.

## 1. Introduction

COVID-19 has created a public health emergency and presently is a global concern. This disease has caused more than 127 million infections and over 2.78 million deaths worldwide as of early 2020 [1]. The disease started in China and ended up across the world. There was a rapid rise in the infection rate during the first 3–4 months of 2020, reaching up to 80%. Nevertheless, the infection rate fell to 65% after implementing global travel restrictions [2]. COVID-19 also spread to different parts of Asia, and the number of infections was very high in Turkey, India, Indonesia, and Iran [3,4,5,6]. The middle to low-income countries in Asia and the Asia-Pacific regions were the worst affected [6]. However, some success stories in controlling the spread of the disease are from small Asian countries such as Taiwan, Vietnam, and South Korea [7]. One of the best examples of the COVID-19 control model is from Taiwan, where only 600 cases and seven deaths were reported in 2020 among a total population of 24 million [7,8,9,10,11]. Other than Asia, a high number of infected patients were recorded in the USA and different countries in Europe [12,13]. Several countries have commenced public health measures to reduce the transmission of the virus. These countries have implemented various policies such as proper lockdown, curfew, and compulsion to wear a mask [14]. However, an effective, safe vaccination program is required to finally curtail the COVID-19 pandemic.

It has been noted that Asian countries have taken various initiatives for the development of the COVID-19 vaccine. Specifically, China and India were the first in this initiative [15,16,17]. Thus, several Chinese and Indian companies are on the frontline to develop a vaccine in Asia [18,19]. The vaccine development started when the Chinese researchers sequenced the SARS-CoV-2 genome. Zhang and his colleagues at Fudan University were the first to sequence the genome of SARS-CoV-2 and submitted it to GenBank, now publicly available at the NCBI database (GenBank) [20]. Utilizing these sequences, our group (India and South Korea) developed an in silico based next-generation vaccine construct for COVID-19 [21]. After that, several similar initiatives were taken for vaccine development. In December 2020, we did an overall survey to understand the scenario of the COVID-19 vaccine development, and we found that 55 vaccine candidates have entered different phases of the clinical trial (27 candidates in the phase-I, 23 candidates in phase-IIl, and 5 candidates in phase-III clinical trial) [22]. Among them, several candidates originated from Asian countries. At present, the COVID-19 vaccination program is in progress throughout the world. The first shot was given when the Moderna Inc. developed mRNA vaccine was approved for clinical trials in the USA in March 2020. In Asia, small clinical trials of this vaccine were started by CanSino Biologics from China just after the Moderna vaccine’s clinical trial in the USA [23].

Till today, several vaccines have been approved for COVID-19 vaccination from Asia. The Asian-origin-approved vaccines are safe and effective. To contain this pandemic, vaccine development was the primary goal right from the beginning of 2020. During the process of vaccine development, different collaborations were made with the lead developers [24,25]. The companies have spent billions of dollars and considerable efforts to develop effective vaccines against SARS-CoV-2. The money came from several governments, private and philanthropic donors [8,26].

Infectious disease outbreaks are natural phenomena occurring from time to time across countries. Vaccination is an effective method to control various infectious diseases, including pandemics [27]. Several countries have taken vaccination programs to prevent contagious diseases [28,29,30]. Presently, the COVID-19 outbreak has made many countries vulnerable both economically and socially [31]. Asia is also facing the same problem. The COVID-19 vaccination is a fight against this pandemic and is essential for the return of the normal living of global society [32,33].

In this manuscript, we discussed current COVID-19 vaccines and vaccination status in Asia. The first section deals with mapping the different approved vaccines originating from Asia, their technological platforms, collaborations during vaccine development, and regulatory approval from other countries of Asian-origin vaccines. In the second section, we tried to analyze the recently started COVID-19 vaccination program. Here, we analyzed the overall vaccination status in Asian countries. In addition, we evaluated the demographic coverage of the COVID-19 vaccination, both the single and double dosage, in some Asian countries. We also calculated cumulative COVID-19 vaccine dosage administered in different Asian countries, COVID-19 vaccine rolling 7-day average in various Asian countries, and COVID-19 vaccine per day doses administrated in selected Asian countries.

Furthermore, we also tried to understand the vaccine efficacy and basic reproduction numbers (R0) to understand the vaccination program’s probable effectiveness. Lastly, in the third section, we explored the factors affecting the COVID-19 vaccination programs in Asia, such as vaccine hesitancy in Asian countries and the cost of the vaccines for low and middle-income countries in Asia. Our analysis will assist in developing effective vaccines and implementing the COVID-19 vaccination program successfully in the Asian region.

## 2. Materials and Methods

### 2.1. Data Collection

We collected the COVID-19 vaccine development and vaccination progress data in Asia from WHO [34]. We also searched the databases such as PubMed [35,36], Web of Science [37], and Google Scholar [38] with different keywords. One example of search terms is “COVID-19 vaccines”, “vaccine hesitancy,” “COVID-19 vaccination,” etc. As a result, we found several relevant information and studies.

We collected data from different other sources such as our world in data (our world in data for COVID-19) [39], Vaccine Tracker (Coronavirus vaccine tracker) [40], Statista (The COVID-19 vaccination Race in Asia) [41], and COVID-NMA [42]. Furthermore, for clinical trial information of the Asian-origin-approved vaccines, we retrieved the data from ClinicalTrials.gov [43]. We also retrieved several data from the Biorender COVID-19 vaccine and therapeutic drugs tracker [44].

### 2.2. Data Analysis and Interpretation

We mapped different approved vaccines originating from Asia, their technological platforms, collaborations during vaccine development, and regulatory approval from other countries of Asian-origin vaccines.

**Figure 1:**
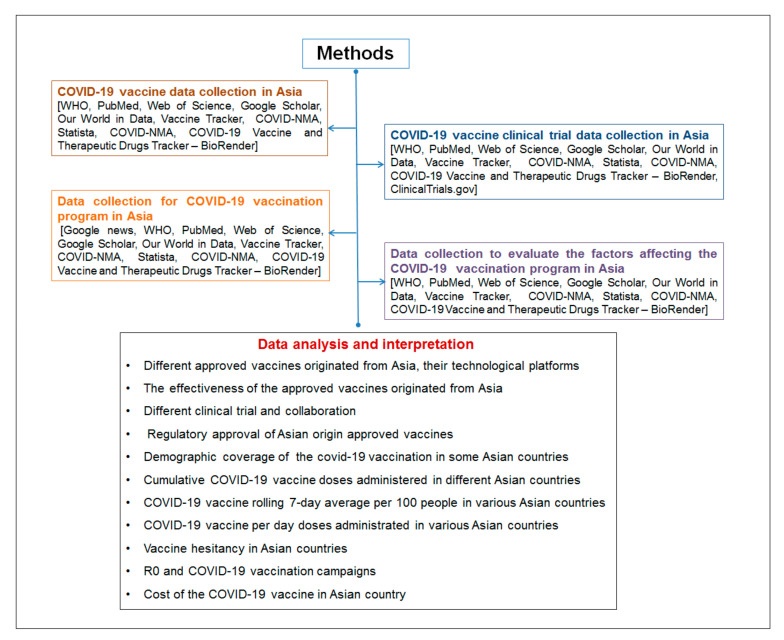
A flowchart describing the methodology of this study.

At the same time, both the single and double doses of vaccination were analyzed in Asian countries. We analyzed the demographic coverage of the COVID-19 vaccination in Asia. The cumulative COVID-19 vaccine doses administered in different Asian countries, the vaccine rolling 7-day average across countries, and the vaccine per day doses administrated in multiple Asian countries were calculated. Different essential statistical tools were used for this analysis. For understanding the overall methodology of this study, a flowchart is presented in Figure 1.

## 3. Results

### 3.1. Different Approved Vaccines Originated from Asia and Their Technological Platforms

**Figure 2:**
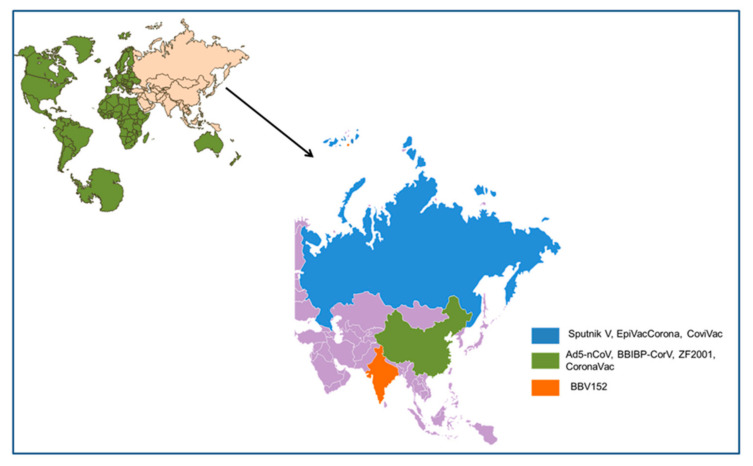
Different Asian-originated COVID-19 vaccines, which received regulatory approval from at least one country and the country of origin.

We found a total of eight approved vaccines that originated from Asia till the end of March 2020 and were approved in at least one country. It includes Ad5-nCoV, BBIBP-CorV (Sinopharm), Sputnik V (Gamaleya), ZF2001 (RBD-Dimer), CoronaVac, EpiVacCorona, BBV152 (Covaxin), and CoviVac. A list for the developer of vaccines, country of origin, the dose details, efficacy, stability, temperature (°C), and clinical trials number is presented in Table 1 and Figure 2.

We listed the technological platform of all the Asian-origin vaccines: conventional inactivated vaccines, viral vector vaccines, modified adenovirus vector-vaccine, and protein subunit vaccines. Four conventional inactivated vaccines that were noted are CoronaVac, BBV152 (Covaxin), BBIBP-CorV (Sinopharm), and CoviVac. Only one viral vector vaccine was observed (Sputnik V (Gamaleya)). Simultaneously, one modified adenovirus vector vaccine (Ad5-nCoV) and two protein subunit vaccines (EpiVacCorona and ZF2001(RBD-Dimer)) were observed (Table 1).

### 3.2. The Effectiveness of the Approved Vaccines Originated from Asia

It is essential to understand the efficacy of the approved vaccines (originated from Asia). The approved vaccines have shown efficacy in clinical trials, and it is essential as it protects both the person and a country’s population [53,54]. In general, it was observed that most of the approved COVID-19 vaccines had efficacy in the range of 70–95% (Table 1). We noted the efficacy of some of the important vaccines, which is as follows: Sputnik V with 91.6% [55], BBIBP-CorV with 79.3% [56],Covaxin with 81% [57].

### 3.3. Different Clinical Trial and Collaboration

**Figure 3:**
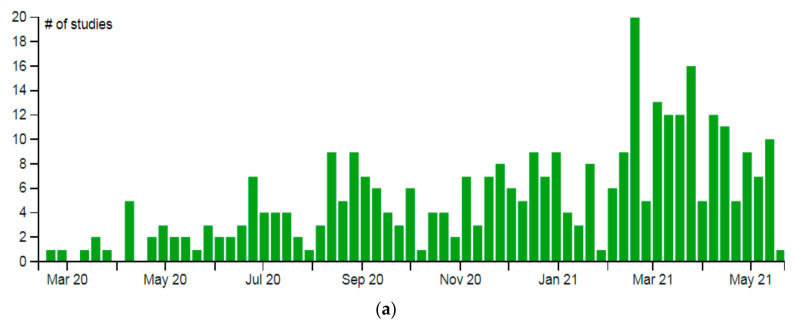

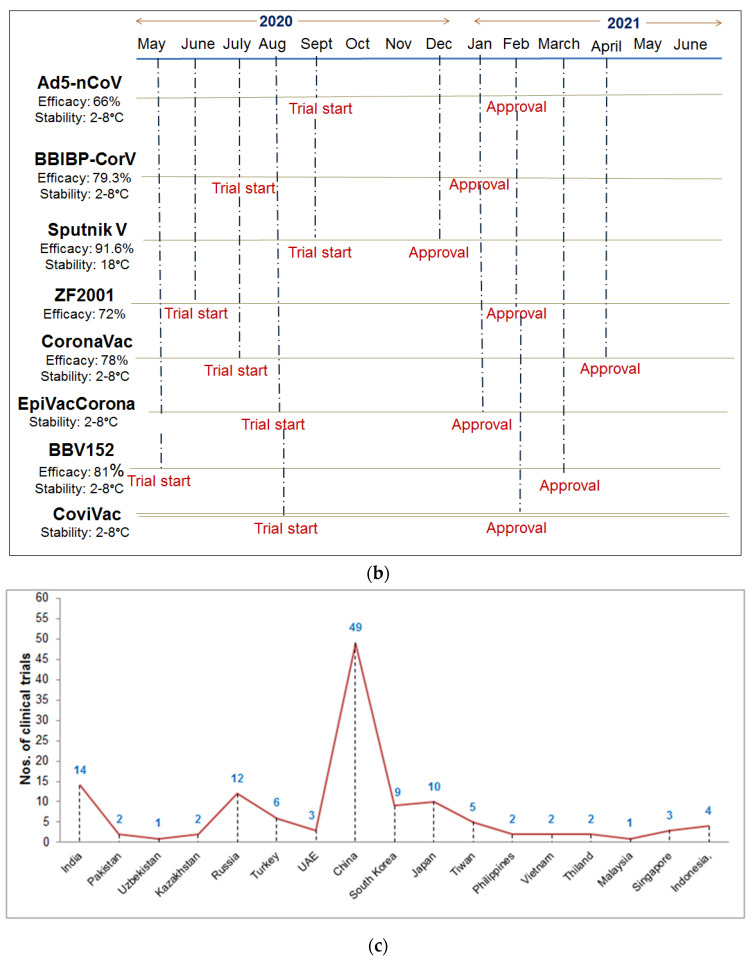
The entire clinical trial landscape of the different COVID-19 vaccines throughout the globe. (**a**) Evaluation report of the total registration of the clinical trials (by a week) mapped from COVID-19 NMA database (https://covid-nma.com, accessed on 25 May 2021) [42]. (**b**) The schematic diagram represents some COVID-19 Asian-origin vaccines. Diagram represent the possible time for the trail to start, date of approval, storage temperature, and efficacy. (**c**) Numbers of clinical trials of Asian-originated COVID-19 vaccines (Vaccine Tracker, Statista, COVID-NMA, ClinicalTrials.gov).

**Figure 4:**
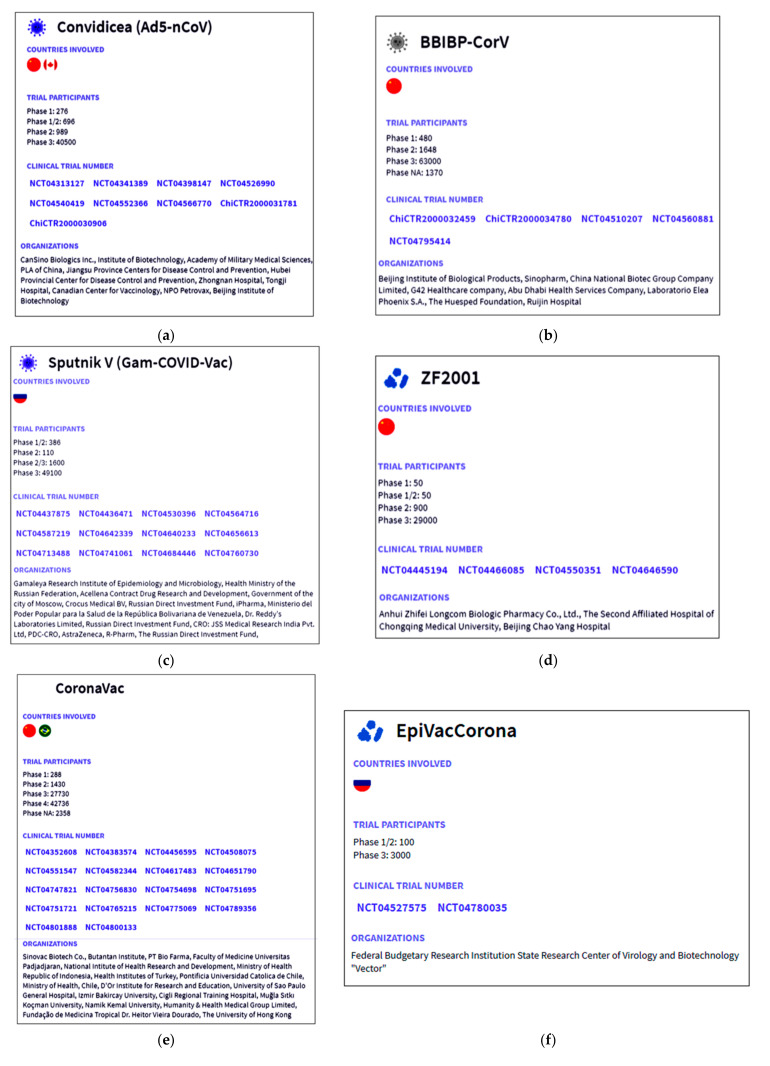

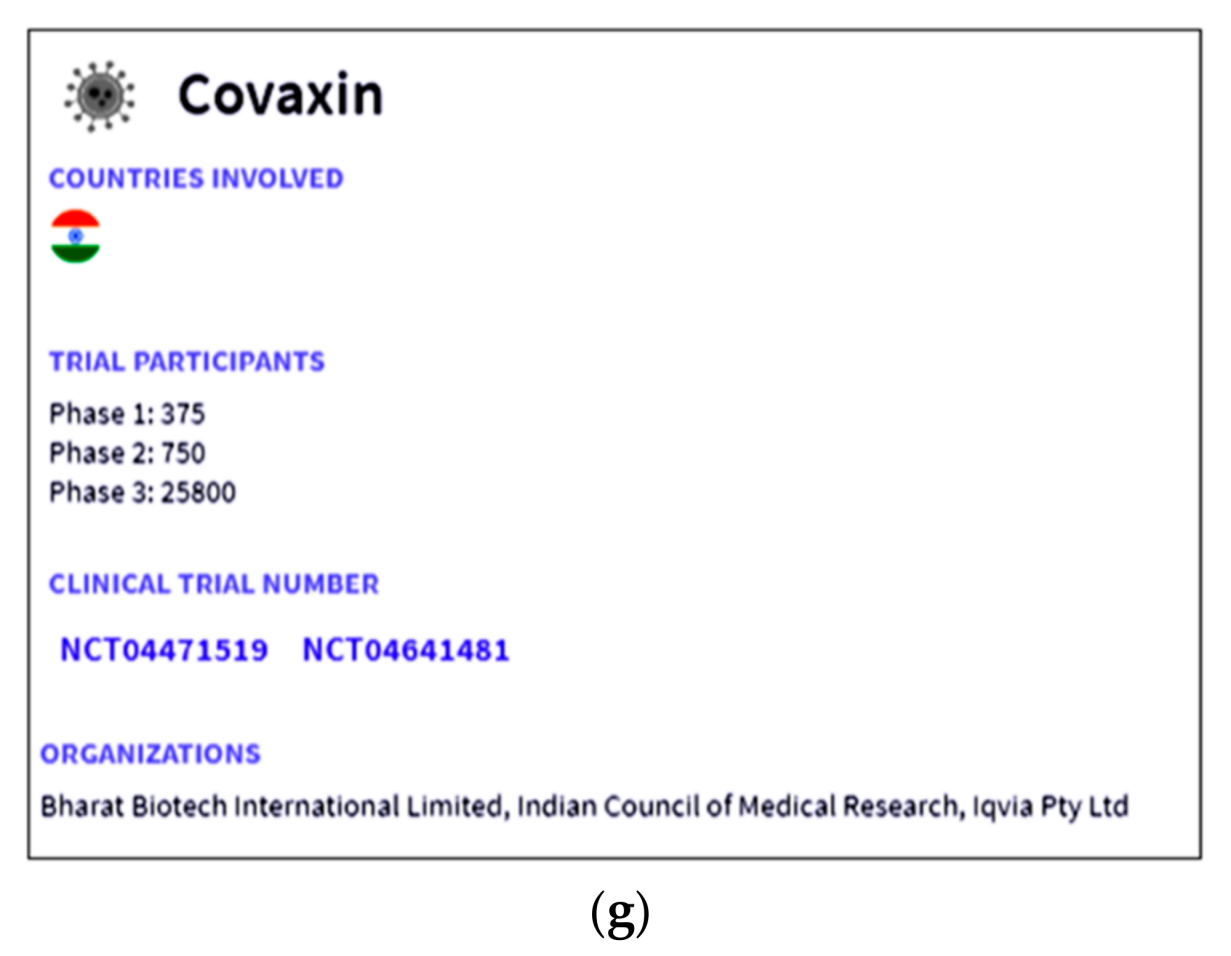
Information about the clinical trials of Asian-origin COVID-19 vaccines such as country involved, trial patients, clinical trial number, and organization involved for a clinical trial or any vaccine development. (**a**) Ad5-nCoV, (**b**) BBIBP-CorV, (**c**) Sputnik V, (**d**) ZF2001, (**e**) CoronaVac, (**f**) EpiVacCorona, and (**g**) Covaxin. (Data source: Biorender).

Immediately after the preclinical stage, most COVID-19 vaccines started their clinical trials (phase-I, -II, and -III) to understand the safety and efficacy. We performed a survey (September–November 2020) and found that during this period, more than 55 vaccine candidates entered the list of clinical trials (source is from ClinicalTrials.gov). As of September–December 2020, we observed 27 vaccine candidates entering the phase-I clinical trials, 23 vaccine candidates entering the phase-II clinical trials, and 5 vaccines entering the phase-III clinical trials, respectively [22]. These trials were registered in the COVID-19 NMA database. We observed a total of 346 clinical trials (throughout the world) as of 21 May 2021. Among them, 246 were randomized, and 100 were nonrandomized clinical studies of COVID-19 vaccines. To this date, 169 clinical trials have recruited patients for the studies. The registration of the clinical trials was analyzed and mapped from the same database and is shown in Figure 3a. Of these, 27 clinical studies have published their data while two were terminated, one was suspended, and one was withdrawn. From the COVID-19 vaccine tracker, we mapped the approval scenario for the Asia-originated vaccines (Figure 3b). The numbers of clinical trials of the Asia-originated COVID-19 vaccines were identified from different countries (Figure 3c) and is recorded, namely Ad5-nCoV (Figure 4a), BBIBP-CorV (Figure 4b), Sputnik V (Figure 4c), ZF2001 (Figure 4d), CoronaVac (Figure 4e), EpiVacCorona (Figure 4f) and Covaxin (Figure 4g).

Different collaborations were made during the development of the vaccines. The significant associations during the development of the Asian-origin vaccines are shown in Table 2. In addition, networking information was collected to understand the collaborations between the collaborating partners. The networking for the partnerships throughout the Asian countries is shown in Table 3.

### 3.4. Regulatory Approval of Asian-Origin Approved Vaccines

**Figure 5:**
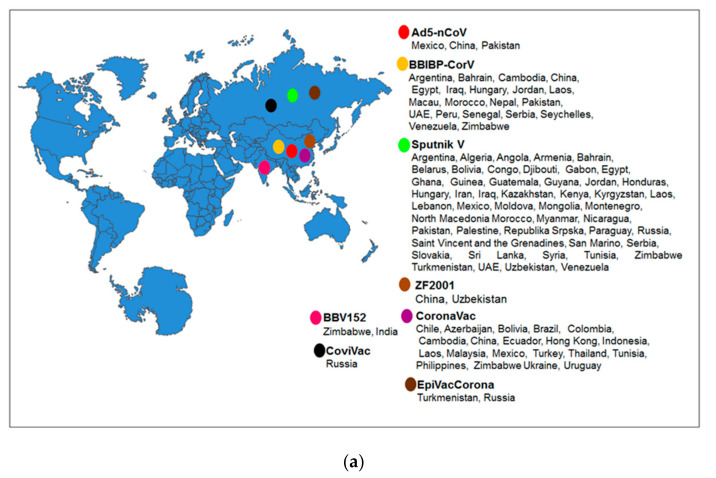

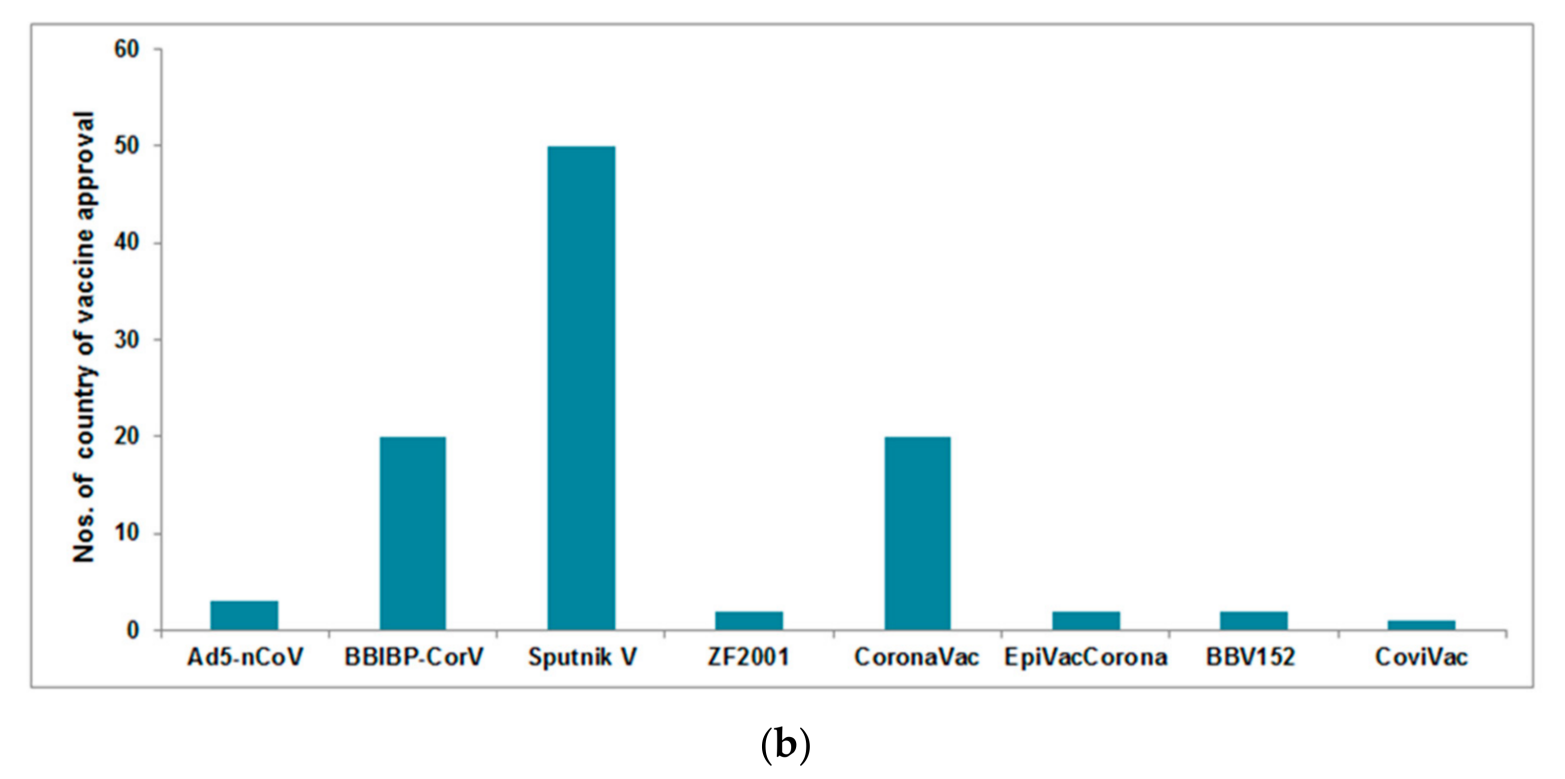
Regulatory approval of Asian-origin COVID-19 approved vaccines. (**a**) Asian-origin COVID-19 vaccines and their approval countries. (**b**) Asian-origin COVID-19 vaccines and number of approval countries. (Data source: Vaccine Tracker, Statista, COVID-NMA, ClinicalTrials.gov, Biorender).

The vaccines approved in various countries in Asia are shown in Figure 5a. All the eight approved vaccines and their approval country number are shown in Figure 5b. Due to the pandemic situation, most vaccines received emergency approval from regulatory authorities in Asia to fight the infection.

### 3.5. Demographic Coverage of the COVID-19 Vaccination in Some Asian Countries

**Figure 6:**
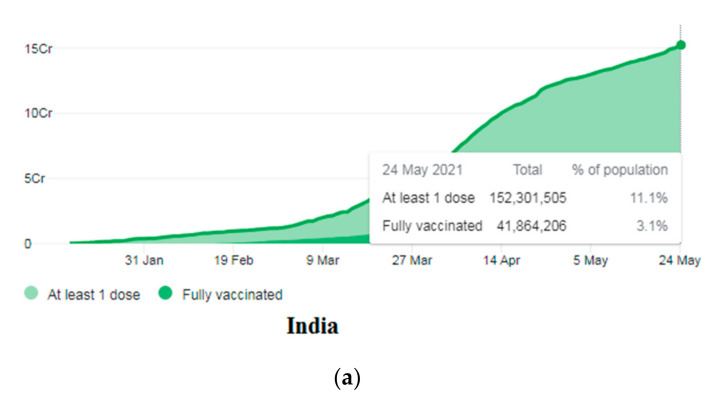

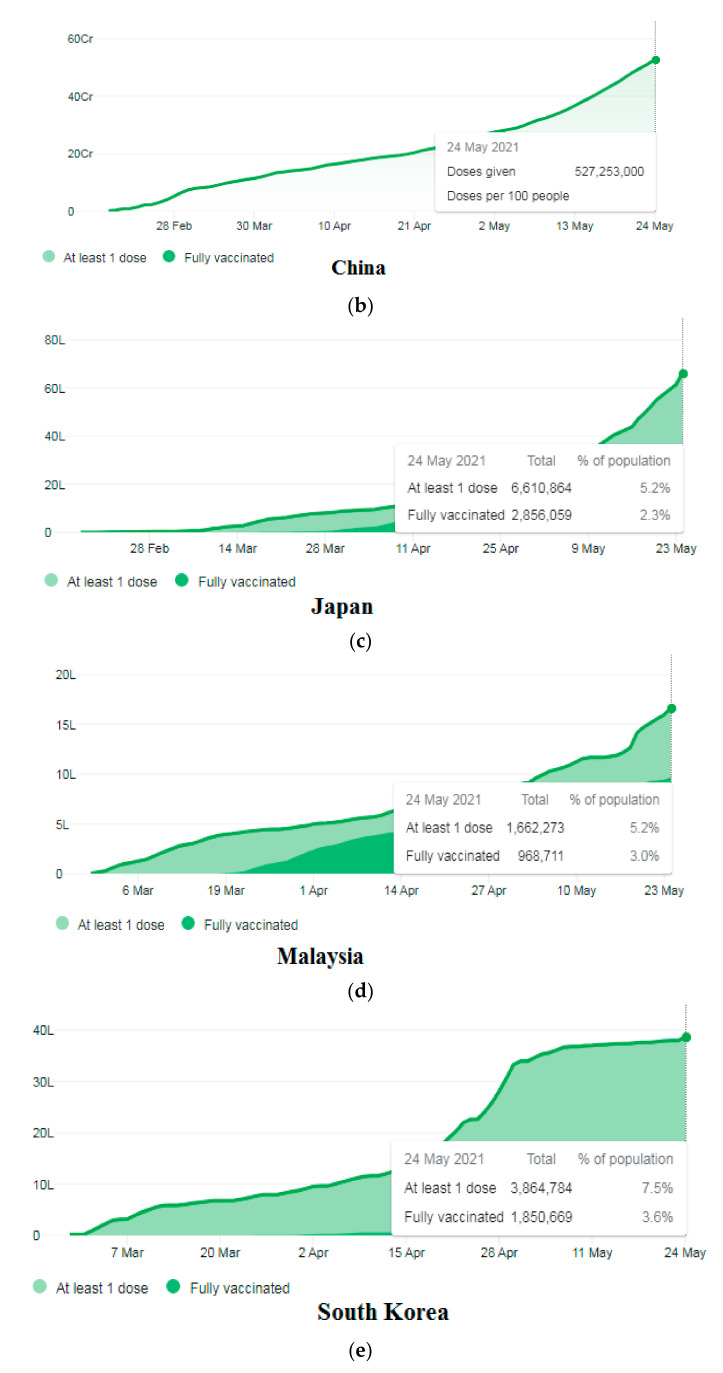

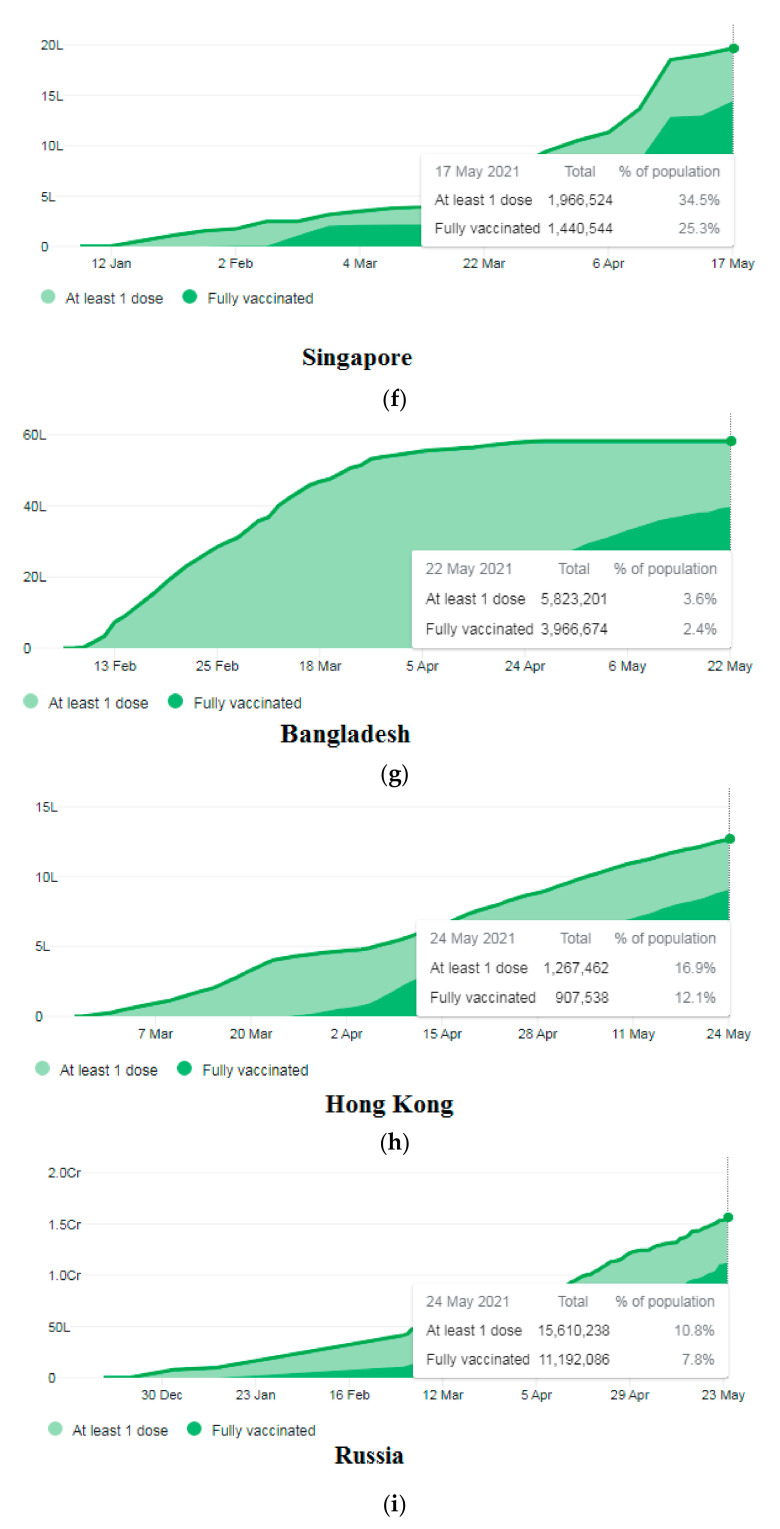
The COVID-19 vaccination status in some Asian countries as of 24 May 2021. (**a**) COVID-19 vaccination status in India. (**b**) COVID-19 vaccination status in China. (**c**) COVID-19 vaccination status in Japan. (**d**) COVID-19 vaccination status in Malaysia. (**e**) COVID-19 vaccination status in South Korea. (**f**) COVID-19 vaccination status in Singapore. (**g**) COVID-19 vaccination status in Bangladesh. (**h**) COVID-19 vaccination status in Hong Kong. (**i**) COVID-19 vaccination status in Russia. (Data source: Our World in Data).

We evaluated the demographic coverage in some Asian countries using the COVID-19 vaccination. The evaluation was regarding the population receiving at least one dose and those who were completely vaccinated. The vaccination report till 24 May 2021 was considered. In total, 11.1% of the total population (152.3 million) received at least one dose in India, and 3.1% of the population is wholly vaccinated (Figure 6a). In China, 527.2 million vaccine doses were given to the people (Figure 6b), and it aims to vaccinate 40% of its total population by the end of July 2021. In Japan, 5.2% of the population has received at least one dose, and 2.3% of the population is completely vaccinated (Figure 6c). In Malaysia, a total of 1.6 million vaccine doses were given to the people (Figure 6d). In South Korea, 7.5% of the population has received at least one dose, and 3.6% is wholly vaccinated (Figure 6e). In Singapore, 34.5% of the people have received at least one dose, and 25.3% are entirely vaccinated (Figure 6f). In Bangladesh, 5.8 million vaccine doses were given to the people (Figure 6g). In Hong Kong, 16.9% of the population has received at least one dose, and 12.1% are wholly vaccinated (Figure 6h). In Russia, 10.8% of the people have received at least one dose, and 7.8% are completely vaccinated (Figure 6i).

**Figure 7:**
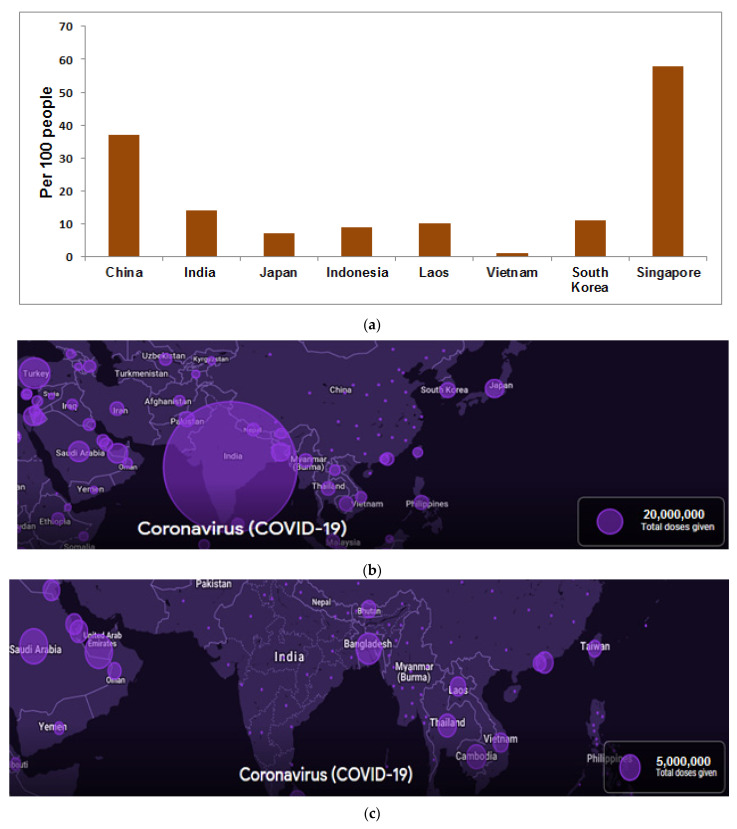
COVID-19 vaccine administration status during COVID-19 vaccination in some Asian countries. (**a**) COVID-19 vaccine administration in some Asian countries (per 100 people in a country’s total population) [41]. (**b**) Asian countries (China and India) which vaccinate the people with more than 20 million vaccine doses) (last 14 days vaccination data from China is not correctly available). (**c**) Asian countries which administrated more than 5 million vaccine doses in the last 14 days to the people (data source: Our World in Data, Google Analytics).

We recorded the COVID-19 vaccine administration in some countries (per 100 people in a country’s total population) in Asia (Figure 7a) and found that China and India have vaccinated over 20 million doses (Figure 7b) (last 14 days, the vaccination data from China is not correctly available). We also found that more than eight countries have administrated above 5 million vaccine doses within 14 days to people (Figure 7c).

### 3.6. Cumulative COVID-19 Vaccine Doses Administered in Different Asian Countries

**Figure 8:**
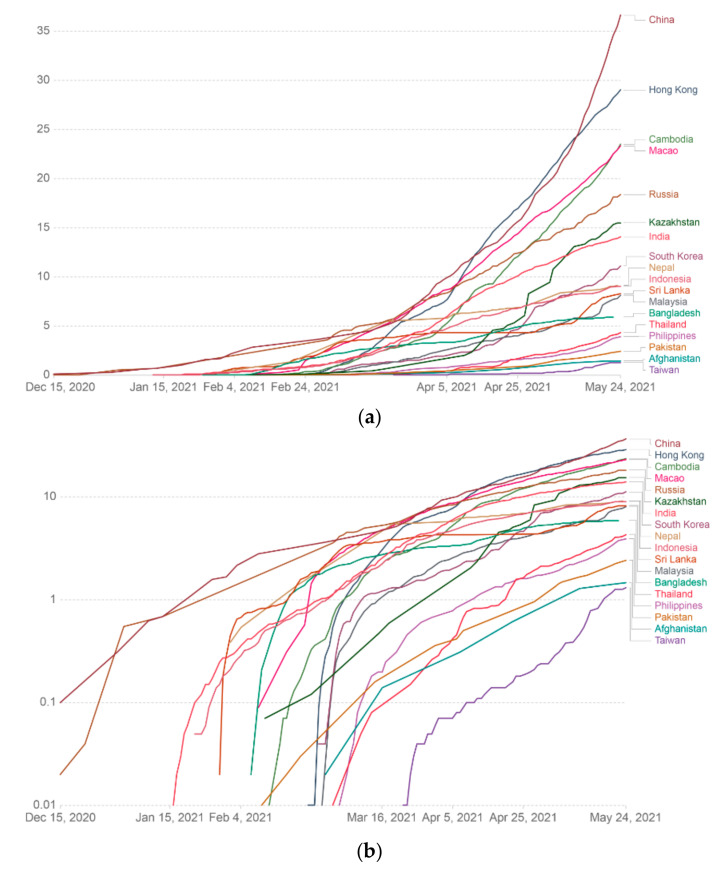

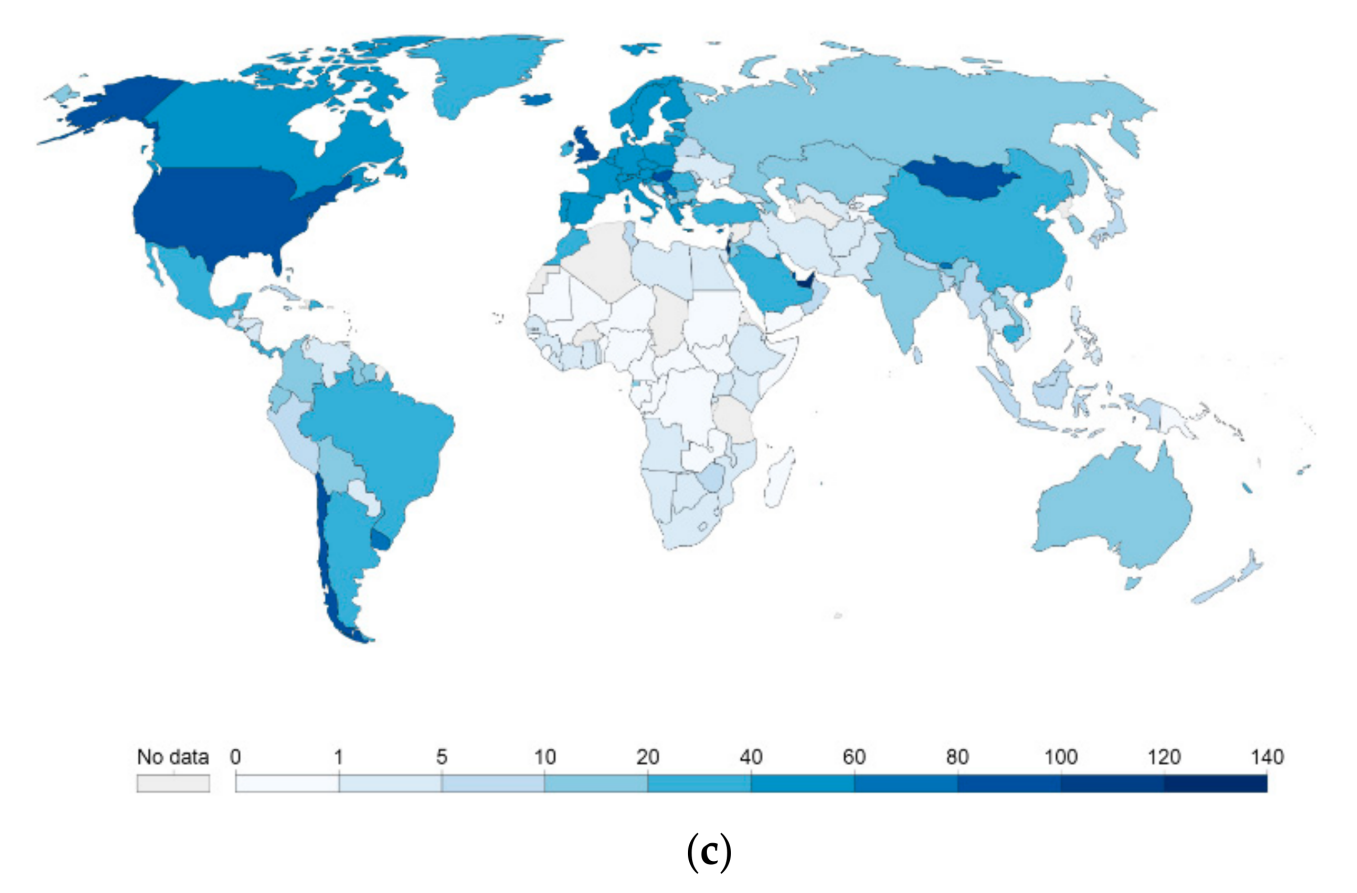
Cumulative COVID-19 vaccine doses administered per 100 people in a country’s total population in different Asian countries. Data counted single-dose vaccine doses issued per 100 people in the total population up to 24 May 2021. (**a**) Cumulative COVID-19 vaccine data (doses administered per 100 people) in different Asian countries are represented through the linear graph. (**b**) Cumulative COVID-19 vaccine data (doses administered per 100 people) in various Asian countries described through the log graph. (**c**) Cumulative COVID-19 vaccine data (doses administered per 100 people) in different Asian countries represented through the Asian map (data source: Our World in Data).

We calculated the cumulative COVID-19 vaccine dose administration in different Asian countries. In this case, we determined the cumulative COVID-19 vaccine in various Asian countries and represented it through a linear graph (Figure 8a). We also calculated the cumulative COVID-19 vaccine in different Asian countries using log graph mode (Figure 8b). Finally, we also represented the cumulative COVID-19 vaccine in various Asian countries using an Asian map and marked it through color variation (Figure 8c).

### 3.7. COVID-19 Vaccine Rolling 7-Day Average Per 100 People in Various Asian Countries

**Figure 9:**
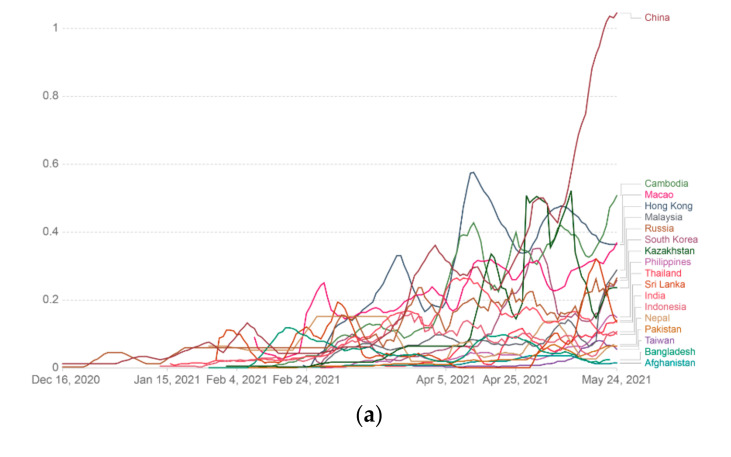

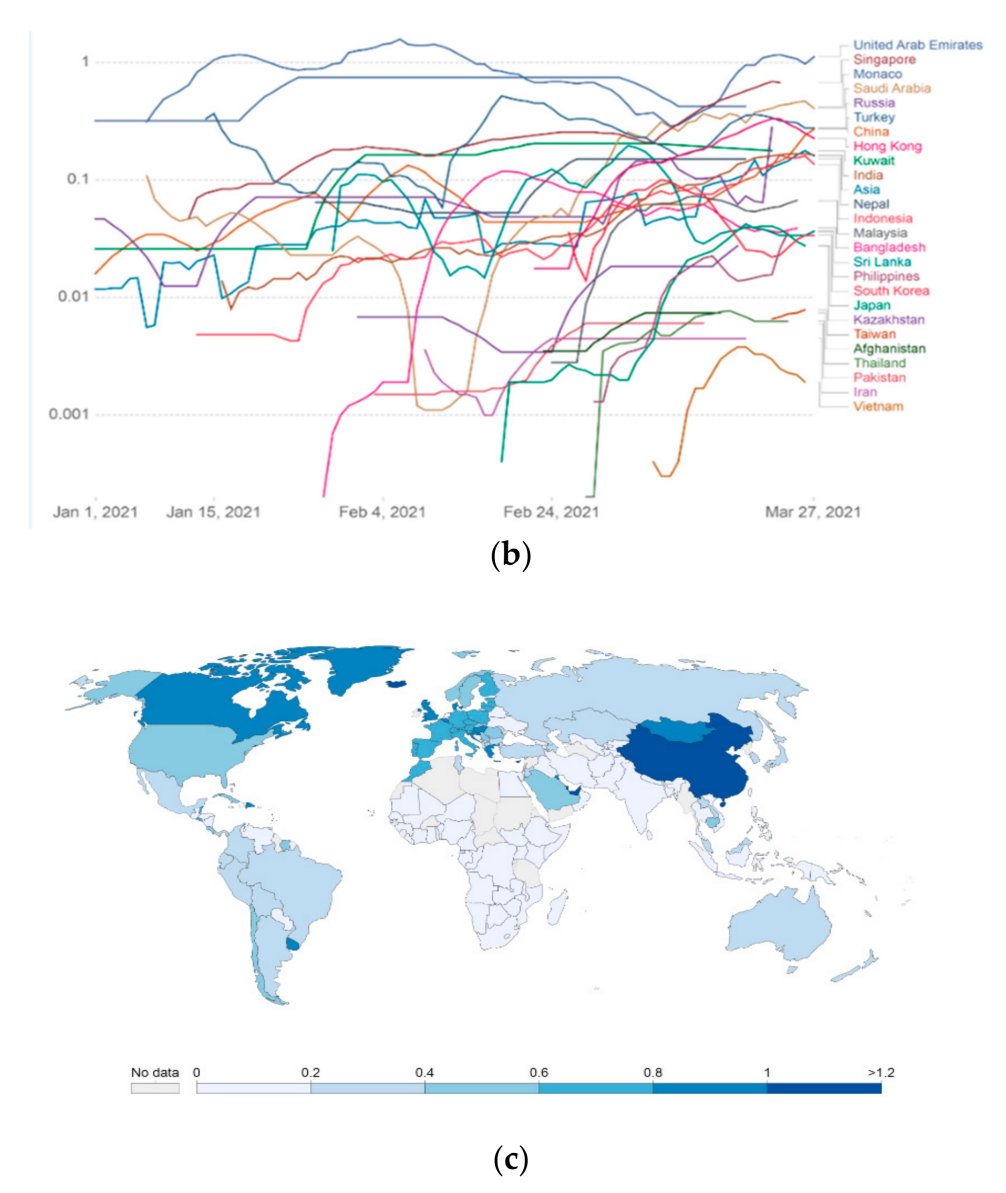
COVID-19 vaccine rolling 7-day average per 100 people in a country’s total population in different Asian countries. Data counted as single-dose vaccine doses administered per 100 people in the total population up to 24 May 2021. (**a**) COVID-19 vaccine rolling 7-day average data (doses administered per 100 people) in different Asian countries represented through a linear graph. (**b**) COVID-19 vaccine rolling 7-day average data (doses administered per 100 people) in various Asian countries represented through the log graph. (**c**) COVID-19 vaccine rolling 7-day average data (doses administered per 100 people) in different Asian countries represented through the Asian map (data source: Our World in Data).

We calculated the COVID-19 vaccine rolling 7-day average per 100 people in various Asian countries and represented it through a linear graph (Figure 9a). The COVID-19 vaccine rolling 7-day average per 100 people in different Asian countries is represented using the log graph mode (Figure 9b). In addition, we also represented the COVID-19 vaccine rolling 7-day average per 100 people in various Asian countries using an Asian map and marked it through color variation (Figure 9c).

### 3.8. COVID-19 Vaccine Per Day Doses Administrated in Various Asian Countries

**Figure 10:**
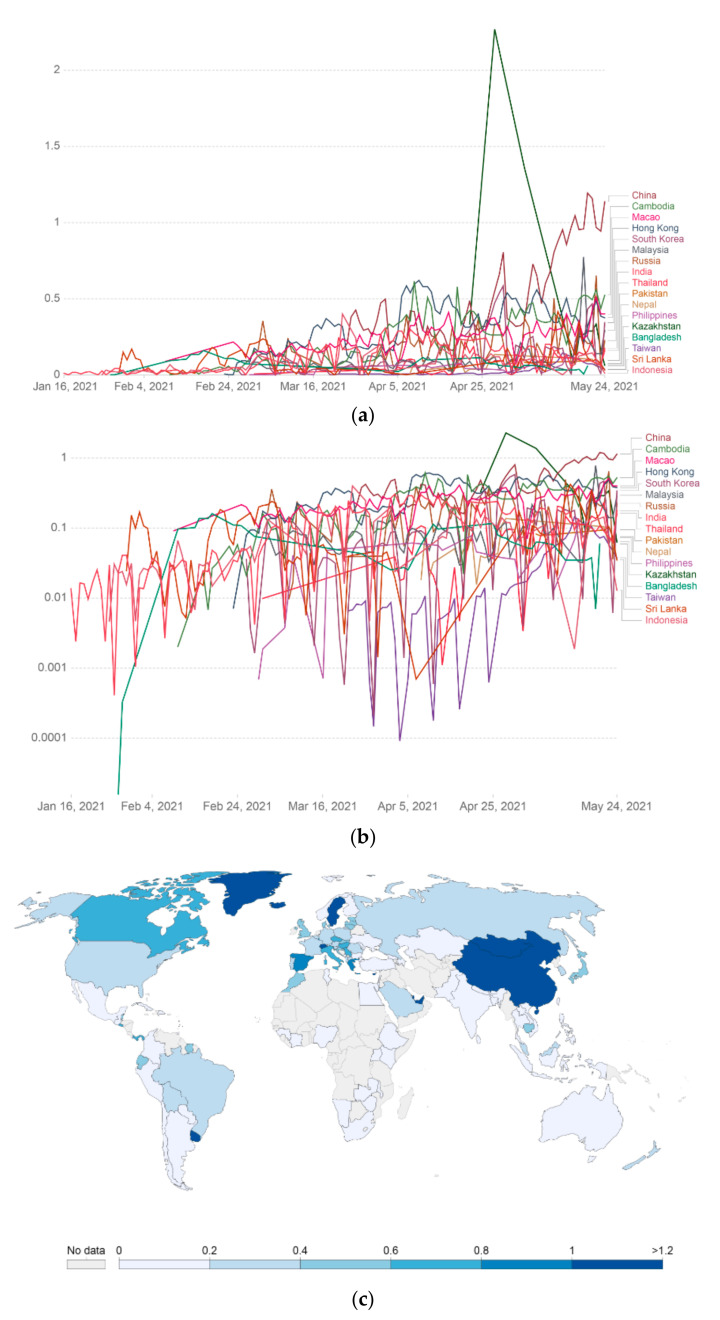
COVID-19 vaccine per day doses administrated average per 100 people in a country’s total population in different Asian countries. Data counted single-dose vaccine doses administered per 100 people in the total population up to 24 May 2021. (**a**) COVID-19 vaccine per day doses administration data (doses administered per 100 people) in different Asian countries represented through a linear graph. (**b**) COVID-19 vaccine per day doses administrated data (doses administered per 100 people) in various Asian countries represented through the log graph. (**c**) COVID-19 vaccine per day doses administration data (doses administered per 100 people) in different Asian countries represented through the Asian map (Data source: Our World in Data).

We also calculated the COVID-19 vaccine per day dose administration in multiple Asian countries and represented it through a linear graph (Figure 10a). Again, in this case, COVID-19 vaccine per day doses administrated in various Asian countries were described using the log graph mode (Figure 10b). Furthermore, we also represented COVID-19 vaccine per day doses administration in multiple Asian countries using an Asian map and marked it through color variation (Figure 10c).

### 3.9. Vaccine Hesitancy in Asian Countries

**Figure 11:**
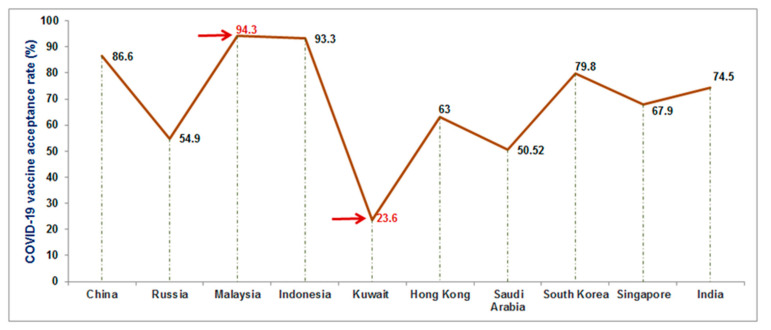
COVID-19 vaccine acceptance rate in some Asian countries. The highest vaccine acceptance rate was noted in Malaysia (94.3%), and the lowest vaccine acceptance rate was reported in Kuwait (23.6%).

Vaccine hesitancy is one of the significant factors that hinder the vaccination drive. It is a global problem for vaccination [58]. Several models have been developed to understand vaccine hesitancy and have been divided into four main categories: The first category is the “vaccine acceptor group” or “vaccine acceptance,” who agreed to take the vaccine. The second category is the “vaccine-hesitant group” or “vaccine-hesitancy,” who may accept vaccination but with considerable concerns about the vaccination. The third category is the “late vaccinators group,” who knowingly or purposefully delay the vaccinating process. The last category is the “rejector group,” which rejects the vaccines [59]. For COVID-19 vaccination, several scientists have reported vaccine hesitancy as a significant problem [60]. Several researchers have tried to understand the COVID-19 vaccine acceptance rates [61,62,63,64]. Lazarus et al. have performed a global survey about the vaccine acceptance rates that varied from country to country. They have demonstrated a range of vaccine acceptance rates from approximately 90% to 55% [65]. We noted vaccine hesitancy in Asian countries such as China, Kuwait, Malaysia, Indonesia, Russian, Hong Kong, Singapore, and India (Table 4). From the reviewed data from the published literature, we observed the highest vaccine acceptance rate in Malaysia (94.3%) and the lowest vaccine acceptance rate in Kuwait (23.6%) (Figure 11). The lowest vaccine acceptance rate was noted due to a lack of awareness of the COVID-19 vaccine and awareness or worry about getting sick due to vaccination. On the other hand, the highest vaccine acceptance rate was observed due to well-organized campaigns and awareness for COVID-19 vaccination. However, most of the surveys were performed using a random sample of the population from every country. Therefore, more COVID-19 vaccination campaigns are required for the lowest vaccine acceptance countries.

### 3.10. Basic Reproduction Number (R0) and COVID-19 Vaccination Campaigns

**Figure 12:**
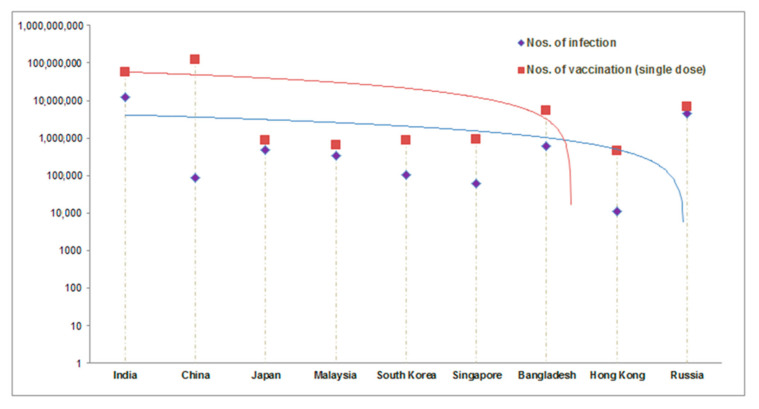
Comparison between the total no of infections and the vaccination status (in a single dose) in some Asian countries. It can help to understand R0 and the requirement of COVID-19 vaccination campaigns. (Data source: Vaccine Tracker, Statista, COVID-NMA, ClinicalTrials.gov, Biorender) [64,70].

Basic reproduction number (R0) is one of the critical factors for COVID-19 vaccination. The R0 metric is used to understand the transmissibility or contagiousness of infective agents. It is an epidemiologic metric [70]. Alimohamadi et al. calculated the mean R0 for COVID-19 through meta-analysis and systematic review from different reported articles. The mean value of R0 was reported as 3.38 ± 1.40. In this analysis, the range of the R0 was noted as 1.90–6.49 [71]. It was pointed out that the pattern of R0 of COVID-19 during the COVID-19 pandemic period and the pattern of R0 influenza during influenza is more or less similar in respect to clinical severity and clinical characteristics [72]. Some Asian countries, such as Taiwan, tried to develop vaccination plans based on their previous preparedness strategies during the influenza pandemic [9]. Farrington has developed a model for R0 and the vaccine efficacy. He has shown a correlation between R0 and the projected effectiveness of a vaccination program (PE), which may help evaluate a vaccination program’s effectiveness. The correlation is PE = 1 − (RV/R0), where PE = projected effectiveness of a vaccination program; RV= the projected reproduction number. This study observed Re(t), an effective reproduction number at time t. The time to time analysis of Re(t) is necessary over time, which is a significant feature of evaluating the vaccination program surveillance [61]. This study plotted the total no of infections and the vaccination progress (in a single dose) in some Asian countries (Figure 12).

### 3.11. Cost of the COVID-19 Vaccines in Asian Countries

**Figure 13:**
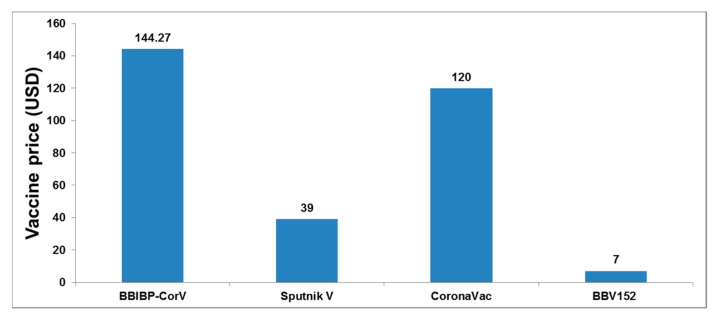
The cost of some Asian-origin COVID-19 vaccines. Presently, the Covaxin vaccine is distributed free from the government hospital for low-income peoples and USD 7 from private hospitals [18,75,76,77].

The vaccine cost is an essential factor for the vaccination program, especially in low- and middle-income countries in Asia. The World Bank report in 2018 showed that 783 million people live in extreme poverty, and 42% of them are located in Asia. We tried to analyze the cost of Asian-origin vaccines (Figure 13) and found that the Indian vaccine production company, the Serum Institute of India, produces the lowest-cost vaccine [73]. The Serum Institute of India has agreed with AstraZeneca to make the Covishield vaccine at a meager cost (USD 3 per dose), accessible by the developing countries across the world [74]. Presently, the Indian vaccines are distributed free from the government hospital for low-income people and USD 7 from private hospitals.

## 4. Discussion

We found that eight COVID-19 vaccines have been approved and originated from Asia, mainly from China, India, and Russia. Four of them are developed by China, one by India, and three by Russia. Furthermore, we observed that these vaccines belong to the four vaccine platforms (conventional inactivated vaccines, viral vector vaccines, modified adenovirus vector-vaccine, and protein subunit vaccines).

Asia is a focal point to conduct clinical trials due to its low cost. The clinical trial in Asia is comparatively 25–40% lower than USA [78,79]. From the vaccine clinical trial point of view, we found the highest number of vaccine clinical trials is going on in China [22]. The second highest number of vaccine clinical trials are going in India [15], and the lowest number of clinical trials are going in Malaysia [1].

Most of the Asian countries have started their COVID-19 vaccination programs. Furthermore, we analyzed the COVID-19 vaccination program. Our evaluation showed that Singapore is the first country to administrate the COVID-19 vaccine per 100 populations among Asian countries (58.24%) (Figure 7a) (41). In addition, we found two Asian countries with 20 million vaccine doses (China and India) (Figure 7b). However, the last 14 days vaccination data from China is not adequately available. Simultaneously, other countries (more than eight) have administrated 5 million vaccine doses to their population in the last 14 days (Figure 7c).

It was observed that Chinese vaccines are sold in several low and middle-income Asian countries. Sinopharm, a Chinese vaccine, has conducted a phase III clinical trial in UAE and reported the vaccine’s efficacy to be 86%. This vaccine is being sent to different countries such as Pakistan [80]. Another Chinese vaccine, CoronaVac, will be sold to other countries such as developing countries like Indonesia and Turkey [80]. The critical Russian vaccine, Sputnik V, was developed by the Gamaleya Research Institute and is being circulated to low and middle-income countries like Vietnam, India, and Nepal [81].

Under the foreign policy, India is delivering the vaccine to the South Asian neighbors. India has donated over 5 million doses of these vaccines to various low-income countries such as Bangladesh, Bhutan, Afghanistan, Maldives, Sri Lanka, and Nepal [75,82].

All the Asian countries have started their COVID-19 vaccination with immense initiative and full efforts in small to medium populated countries and low to medium-income countries. However, vaccination programs are suffering from several unavoidable factors that hamper their progress, such as the cost of the COVID-19 vaccines, vaccine hesitancy, and vaccine illiteracy. Wouters et al. discussed several challenges for the global access to COVID-19 vaccines. They have highlighted the significant difficulties such as price, affordability, acceptance of COVID-19 vaccines, logistical and administrative challenges, storage requirements, and production capacities [76]. Thus, in this domain, more vaccination campaigns and low-cost COVID-19 vaccines must support low to medium-income Asian countries to build up public trust and confidence in COVID-19 vaccines.

## 5. Conclusions

Fortunately, several COVID-19 vaccines have been produced within a year, and the vaccination programs have started across several countries to stop the pandemic. However, at the same time, significant planning and good execution are required to vaccinate the entire population of each country to end the pandemic.

Nevertheless, several essential questions still need to be resolved by the researchers. One of them is to evaluate the durability of the protective immune responses of the COVID-19 approved vaccines. At the same time, several mutated variants have originated in recent times, which include B.1.351 lineage (South Africa), B.1.1.7 lineage (United Kingdom), and P.1 lineage (Brazil). Currently, these variants are circulating throughout Asia. Therefore, can the existing COVID-19 vaccines generate the protective immune responses for the newly mutated variants, or do we need to develop new vaccines? Therefore, for the betterment of the human race, we need to resolve these questions quickly.

However, our analysis will advance the understanding of the vaccination program taken by different Asian countries to improve future vaccination strategies.

## Figures and Tables

**Table 1:** Approved Asian-origin COVID-19 vaccines and their developers, dose, efficacy, stability, technological platforms, and clinical trials number.

Sl. No.	Name of Vaccines	Developer of the Vaccines	Country of Origin	Dose	Efficacy	Stability (Temperature °C)	Technological Platforms	Clinical Trials No	References
1.	Ad5-nCoV	Beijing Institute of Biotechnology, CanSino Biologics	China	Single dose	66%	2–8	Modified adenovirus vector vaccines	NCT04380701, NCT04523571, NCT04368728, NCT04368728	[45]
2.	BBIBP-CorV (Sinopharm)	China National Pharmaceutical Group Corporation, Beijing Institute of Biological Products, Wuhan Institute of Biological Product	China	Double doses (four to three weeks interval)	79.3%	2–8	Conventional inactivated vaccines	NCT04560881	[46]
3.	Sputnik V (Gamaleya)	Gamaleya Research Institute of Epidemiology and Microbiology	Russia	Double doses (three weeks interval)	91.6%	18	Viral vector vaccines	NCT04436471, NCT04437875, NCT04530396	[47]
4.	ZF2001 (RBD-Dimer)	Chinese Academy of Sciences, Anhui ZhifeiLongcom Biologic Pharmacy Co. Ltd.	China	Triple doses (30 days interval)	72%	-	Protein subunit vaccines	NCT04646590	[48]
5.	CoronaVac	Sinovac Biotech Ltd.	China	Double doses (two weeks interval)	78%	2–8	Conventional inactivated vaccines	NCT04551547, NCT04383574, NCT04352608, NCT04617483, NCT04582344, NCT04508075	[49]
6.	EpiVacCorona	State Research Center of Virology and Biotechnology VECTOR	Russia	Double doses (four weeks interval)	-	2–8	Protein subunit vaccines	NCT04527575, NCT04780035	[50]
7.	BBV152 (Covaxin)	Indian Council of Medical Research (ICMR), and Bharat Biotech Ltd.	India	Double doses (four weeks interval)	81%	2–8	Conventional inactivated vaccines	NCT04641481, NCT04471519	[51]
8.	CoviVac	Russian Academy of Sciences	Russia	Double doses (two weeks interval)	-	2.8	Conventional inactivated vaccines	NCT04619628	[52]

**Table 2:** Different stages of collaboration between the collaborators involved in the development of COVID-19 vaccines. This table provides information about the COVID-19 vaccines from Asia and approved by at least one country.

Sl. No.	Name of Vaccines	Developer of Vaccines	
		Collaborator (1st)	Collaborator (2nd)
1.	Ad5-nCoV	Beijing Institute of Biotechnology (Beijing, China)	CanSino Biologics (Tianjin, China)
2.	BBIBP-CorV	China National Pharmaceutical Group Corporation, Beijing Institute of Biological Products (Beijing, China)	Wuhan Institute of Biological Products (Wuhan, China)
3.	Sputnik V	Gamaleya Research Institute of Epidemiology and Microbiology (Moscow, Russia)	Dr. Reddy India (to conduct the clinical trials and distribution of the vaccine in India)
4.	ZF2001	Chinese Academy of Sciences (Beijing, China)	Anhui ZhifeiLongcom Biologic Pharmacy Co. Ltd. (Beijing, China)
5.	CoronaVac	Sinovac Biotech Ltd. (Beijing, China)	Instituto Butantan, Brazil
6.	EpiVacCorona	State Research Center of Virology and Biotechnology VECTOR (Novosibirsk Oblast, Russia)	Not available
7.	BBV152	National Institute of Virology, Indian Council of Medical Research (Pune, India)	Bharat Biotech International Limited (Hyderabad, India)
8.	CoviVac	Chumakov Centre, Russian Academy of Sciences (Moscow, Russia)	Not available

**Table 3:** Networking to understand the collaboration between the partner country involved in clinical trials or any point of COVID-19 vaccine development.

Scheme	Country Name	No. of Clinical Trials	Vaccine Clinical Trials Partner Country
1.	India	15	ARG, CHL, COL, CZE, DEU, ESP, FRA, ITA, NLD, PER, SWE, USA
2.	Nepal	1	PEL, PRA, COL, DEU, DOM, PAN, PHL, POL, JAF
3.	China	59	ECU, IDN, PAK, UZB
4.	South Korea	9	-
5.	Japan	13	-
6.	Taiwan	6	VNM
7.	Hong Kong	2	-
8.	Vietnam	3	TWN
9.	Thailand	3	-
10.	Singapore	3	USA
11.	Indonesia	4	CHN, ECU, PAK, UZB, BRA
12	Malaysia	1	BRA
13.	Philippines	2	BEL, BRA, COL, DEU, DOM, ESP, FRA, GBR, NPL, PAN, POL, USA, GAF
14.	Pakistan	3	ARG, CHL, CHN, ECU, IDN, MEX, RUS, UZB
15.	United Arab Emirates	3	BHR, EGY, JOR
16.	Bahrain	1	ARE, EJY, JOR
17.	Azerbaijan	1	-
18.	Uzbekistan	1	CHN, ECU, IDN, PAK
19.	Kazakhstan	2	-
20.	Russia	13	ARG, BLR, CHL, MEX, PAK

**Table 4:** COVID-19 vaccine acceptance rate in different Asian countries as observed from various studies.

Sl No.	Countries	COVID-19 Vaccine Acceptance Rate	Remark	References
1.	China	88.6%	Survey performed in general population (*n* = 712)	[65]
2.	Russia	54.9%	Survey performed in general population (*n* = 680)	[65]
3.	Malaysia	94.3%	The survey performed in male population (*n* = 1159)	[66]
4.	Indonesia	93.3%	The survey performed in general population (*n* = 1359)	[67]
5.	Kuwait	23.6%,	The survey performed in male population (*n* = 771)	[68]
6.	Hong Kong	63.0%	The survey performed in nurse population (*n* = 1205)	[69]
7.	Saudi Arabia	50.52%	The survey performed in healthcare workers population (*n* = 673)	[61]
8.	South Korea	79.8%	The survey performed in general population (*n* = 752)	[61,65]
9.	Singapore	67.9%	The survey performed in general population (*n* = 655)	[61,65]
10.	India	74.5%	The survey performed in general population (*n* = 742)	[61,65]

## Data Availability

Not applicable.
